# Overexpression of Kynurenine 3-Monooxygenase Correlates with Cancer Malignancy and Predicts Poor Prognosis in Canine Mammary Gland Tumors

**DOI:** 10.1155/2019/6201764

**Published:** 2019-05-02

**Authors:** Yi-Han Chiu, Han-Jung Lei, Kuo-Chin Huang, Yi-Lin Chiang, Chen-Si Lin

**Affiliations:** ^1^Department of Nursing, St. Mary's Junior College of Medicine, Nursing and Management, Yilan 26647, Taiwan; ^2^Institute of Long-Term Care, Mackay Medical College, New Taipei City 25245, Taiwan; ^3^Graduate Institute of Veterinary Clinical Science, School of Veterinary Medicine, National Taiwan University, Taipei 10617, Taiwan; ^4^Holistic Education Center, Mackay Medical College, New Taipei City 25245, Taiwan; ^5^Animal Cancer Center, College of Bioresources and Agriculture, National Taiwan University, No. 1, Sec. 4, Roosevelt Road, Taipei 10617, Taiwan

## Abstract

Tumor biomarkers are developed to indicate tumor status, clinical outcome, or prognosis. Since currently there are no effective biomarkers for canine mammary tumor (CMT), this study intended to verify whether kynurenine 3-monooxygenase (KMO), one of the key enzymes involved in tryptophan catabolism, is competent for predicting prognosis in patients with CMT. By investigating a series of 86 CMT clinical cases, we found that both gene and protein expression of KMO discriminated malignant from benign CMTs and was significantly higher in stage IV and V tumors than in lower-stage CMTs. About 73.7% of malignant CMTs showed strong expression of KMO which correlated with lower overall survival rates in patients. Further, downregulation of KMO activity significantly inhibited cell proliferation of CMT cells. Taken together, the findings indicated that KMO is a potential biomarker for tumor diagnosis, and this might open up new perspectives for clinical applications of CMT.

## 1. Introduction

Dogs are viewed as a desirable animal model for human cancer research, as they share a living environment closely related to humans, with similar development patterns of spontaneous tumors and cancer epidemiology [1]. In addition, the genes associated with cancer are much more closely related between dogs and humans than between mice and humans [2]. Recently, many studies have highlighted the similar risk factors are associated with breast cancer among dogs and humans. For example, the outbred nature of dogs compared with mice provides a similar level of genetic diversity among dogs as that found among humans [1]. The* BRCA *gene acts as the tumor suppressor and is significant for the development of canine mammary tumors (CMTs) and human breast cancer [3]. Studies demonstrated that BRCA 1/2 made approximately equal contributions to early-onset human breast cancer, and higher prevalence of BRCA gene mutation was found in breast cancer patients from China and England to show its relevance in the development of breast cancer [4, 5]. Moreover, inbreeding traits within particular breeds of dogs result in low genetic variation [6], which may also aid the identification of potential risk factors or biomarkers for both human and canine cancer malignancy.

Biomarkers are useful tools in cancer diagnosis, tumor monitoring, and prognosis. Most biomarkers are involved in tumor development and therefore can be applied in cancer therapies [7–10]. Estrogen receptor (ER), progesterone receptor (PR), and human epidermal growth factor receptor 2 (HER2) are biomarkers measured in routine examinations for human breast cancer. The progression of breast cancer and canine mammary tumors is mainly stimulated by hormones. In human breast cancer, patients with tumors that are ER-positive and/or PR-positive have a better response to hormonal treatment and there is a lower risk of mortality after diagnosis as compared with patients with ER-negative and/or PR-negative tumors [11]. In canine mammary tumors, studies have demonstrated that the expression of ER-*α* or PR is related to the histological subtype of canine mammary tumors [12], the occurrence of metastases [13], and the survival rate [14–16], but some studies showed no correlations between these factors [17, 18]. HER-2 is a cell membrane surface-bound receptor tyrosine kinase that is involved in several signal transduction pathways and promotes cell growth. Protein overexpression or gene amplification of HER-2 in breast cancer often correlates with poorer clinical outcomes [19–22], and therefore HER-2 is used as an indicator for prognosis [20, 23–25]. The role of HER-2 overexpression in canine mammary tumors is still controversial. Some studies have demonstrated that a high level of HER-2 protein is related to poorer outcomes, such as a higher tumor grade or a greater mitotic count [26, 27], while other studies have demonstrated opposite results [28, 29]. Although other molecular markers have shown potential for diagnosis and prognosis, there is no sufficient evidence proving their efficacy for routine examination and treatment [30–32]. Therefore, it is important to discover new potential biomarkers for clinical application for canine mammary tumor therapy.

Kynurenine 3-monooxygenase (KMO) is a key enzyme in the kynurenine pathway. KMO catalyzes the hydrolysis of kynurenine (KYN) to form 3-hydroxy kynurenine (3-HK) and further generates the downstream metabolite quinolinic acid. Both 3-HK and quinolinic acid may lead to excitotoxicity in the CNS and act as important factors in neurodegenerative diseases [33–36]. As KMO is located at the critical branching point in the kynurenine pathway, elevation of KMO protein shifts the pathway towards the formation of 3-HK instead of kynurenine acid, which is an antagonist of NMDA receptors to protect neuronal cells from the excitotoxicity. KMO plays a role in balancing NMDA receptor agonists and antagonists; therefore, KMO inhibitors can be applied in therapy for neurodegenerative diseases [37]. Presently little is known about KMO for its significance on tumor development. Jin et al. found that high KMO expression is correlated with aggressive malignant phenotype of human hepatocellular carcinoma (HCC) cells and poor prognosis and thus concluded that KMO can be served as a promising biomarker of HCC prognosis [38]. A high level of KMO promotes the synthesis of downstream metabolites of the kynurenine pathway, such as 3-HK, 3-hydroxyanthranilic acid, and quinolinic acid, which participate in the regulation of the immune response and tumor tolerance [39, 40]. On the other hand, a high level of quinolinic acid might stimulate more NMDA receptors, which promotes tumor proliferation through the ERK pathway.

No other study has investigated the role of KMO in canine tumor development. In this study we disclose the association between KMO expression and the malignancy of canine mammary tumors. This study aimed to verify whether KMO is a potential biomarker for the diagnosis of CMT and whether KMO can be a useful molecule in prognostic prediction and therapeutic development for mammary tumors in the future.

## 2. Materials and Methods

### 2.1. Canine Tissue Specimens

Canine mammary tumor tissue specimens were collected in accordance with regulations of the Institutional Animal Care and Use Committee (IACUC) at National Taiwan University Veterinary Hospital and conformed to the guidelines of the protocol IACUC-NTU-101-EL-106. The patients were diagnosed and underwent surgery to remove tumors from 2012 to 2016. All of the patients underwent surgical removal of CMT without other therapy. The clinical histories of the patients were recorded in depth, and follow-up information was continually documented until May 2018. The histological classification and stage of CMT were determined according to the guidelines of the World Health Organization [41]. All tumor pathological diagnoses in this study were done before analyzing the role of KMO in CMT malignancy, but the blind tests were performed by our operators to investigate the KMO gene and protein expression of the tumor cases.

### 2.2. Real-Time RT-PCR

Total RNA was extracted from collected CMT specimens using TRIzol (Invitrogen) and treated with DNase I (Fermentas) to remove contaminated genomic DNA for real-time RT-PCR analysis. Reverse transcription was carried out using a Mastercycler Personal thermal cycler (Eppendorf) with SuperScript II RT (Invitrogen) to synthesize complementary DNA. Primers that specifically bind to canine indoleamine 2,3-dioxygenase (*IDO*) and* KMO* genes were designed using Primer Express software (Applied Biosystems). The housekeeping genes used were *β*-actin and hypoxanthine-phosphoribosyl transferase (*HPRT*), which represents one of the best reference genes for canine mammary gland [42] (Table 1). Real-time RT-PCR was performed on a Bio-Rad real-time PCR machine with the use of SYBR Green PCR Master Mix according to the procedure described previously [43]. Data were presented as fold change in gene expression level in the sample normalized to the housekeeping genes using 2^-ΔCt^ method.

### 2.3. Immunohistochemistry and Protein Scoring System

Sections (5-*μ*m-thick) of formalin-fixed, paraffin-embedded tumor specimens were deparaffinized by submerging slides in two changes of xylene for 20 min each time. Fresh xylene was used for the second tank. The sections were then rehydrated in graded ethanol for 5 min each. After rehydration, the sections were rinsed with distilled water and antigen retrieval was performed with citrate buffer (10.2 mM Trisodium citrate dihydrate, 1.9 mM Citric acid hydrate, pH 6.0) in a decloaking chamber (BIOCARE MEDICAL) at 121°C for 3 min and then at 90°C for 30 s. Endogenous peroxidase activity was quenched using 3% hydrogen peroxide in PBS for 30 min at room temperature, and then the slides were rinsed with Tris-buffered saline (TBS, 24.7 mM Tris-base, 136.9 mM Sodium chloride, pH 7.6) and were blocked with 3% bovine serum albumin (BSA) in TBS for 1 h at room temperature. After blocking, the slides were incubated with rabbit anti-human KMO polyclonal antibody (Proteintech) at a 1:50 dilution in blocking buffer. The rabbit anti-human KMO polyclonal antibody was pretested on human and dog's kidneys as a positive control. Rabbit normal serum (Biogenex) replaced the primary antibody in the same protocol as a negative control. All of the slides were incubated with the primary antibodies overnight at 4°C. On the next day, the slides were rinsed with TBS buffer and the signals of proteins were detected by BioGenex Super Sensitive™ Detection Systems (BioGenex). Briefly, the slides were incubated with Super enhancer™ and Polymer-HRP (BioGenex) for 1 h each at room temperature. TBS buffer was used to wash the slides following each staining step. The slides were treated with Diaminobenzidine tetrahydrochloride (DAB) (BioGenex), which was used as a substrate to visualize protein signals for 1 min and then stained with Mayer's hematoxylin (Sigma-Aldrich) for 30 s. The sections were washed with distilled water for 10 min and then dried at room temperature. After dehydration, the slides were mounted by water-soluble glycerol gelation and examined under a bright-field microscope (Olympus).

All of the immunohistochemical slides were examined by a veterinary pathologist who did not have the patients' clinical information. A total of 5 random fields were chosen from tumor regions to evaluate the expression of each protein. The IHC staining of the samples was evaluated by a gynecological histopathologist using the immunoreactive scoring (IRS) system as described previously [44]; the system is used to rank the protein expressions and the value that equals the staining intensity multiplied by the percentage of positive cells [45]. Grading was performed in a blinded fashion. Samples were interpreted as COX-2-positive if the IRS was ≥4. The standard IRS scores are shown in Table 2. The level of KMO protein was examined under high-power microscopic fields (HPFs, 400×) and scored by the IRS system. The standard for staining intensity is shown in Figure 3.

### 2.4. Assays for Verifying KMO Biofunctions

Canine CMT cell lines CMT-1 and MPG were kindly provided by Dr. Lin CT of the School of Veterinary Medicine, National Taiwan University (Taipei, Taiwan). Both were cultured in Dulbecco's modified Eagle's Medium (DMEM, Gibco) supplemented with 10% fetal bovine serum (FBS, Caisson) and 1% penicillin/streptomycin (Caisson) at 37°C in a humidified atmosphere of 5% CO_2_. To verify the role of KMO in cell growth, 3000 cells/well of CMT-1 or MPG cells were seeded in a 96-well plate and treated with KMO inhibitor, Ro 61-8048 (Sigma-Aldrich), at the indicated concentrations for 24, 48, and 72 h. After the treatment, quantification of cell proliferation was performed using WST-1 reagent according to the manufacturer's protocol (Roche). For* KMO* knockdown, small interfering RNAs (siRNAs), including control and* KMO*, were used, and the reagents were all purchased from Santa Cruz Biotech Inc. CMT-1 and MPG cells were transfected for 48 h with siRNAs against* KMO *(Forward 5′-CCAAGGUAUUCCCAUGAGATT-3′, reverse 5′-UCUCAUGGGAAUACCUUGGTT-3′; scramble siRNA duplex: forward: 5′-UUCUC CGAACGUGUCACGUTT-3′; reverse: 5′-ACGUGACACGUUCGGAGAATT-3′). The cell viability of the cells was quantified using WST-1 and cell extracts were analyzed by KMO immunoblotting.

### 2.5. Western Immunoblotting

The sample (30 *μ*g of protein/lane) was subjected to SDS-PAGE and blotted from 12% (w/v) polyacrylamide gel to a hydrophobic polyvinylidene difluoride (PVDF) membrane for WB analysis. After blocking the PVDF membrane in PBS, 0.05% Tween 20 (PBST) plus 5% skim milk for 2h, the membrane was then sequentially incubated with the anti-human KMO polyclonal antibody (1:2000) (Proteintech) for 2h, and horseradish peroxidase conjugated anti-rabbit IgG (A9169, Sigma-Aldrich) for 1 h at room temperature. Finally, the membrane was washed extensively with PBST and developed with a chemiluminescent peroxidase substrate (Sigma-Aldrich).

### 2.6. Statistical Analysis

Comparisons of mean values were performed using independent two-sample* t* tests with SPSS 16.0 statistics software. The associations between the variables of the categorical factors, including clinical outcomes and the expression of proteins, were calculated by Spearman's correlation coefficient. The significance of the difference between the variables of the categorical factors was determined using a two-tailed *χ*^2^ test. The Kaplan–Meier method was used to estimate the survival durations through the follow-up period.

## 3. Results

### 3.1. KMO Gene Expression and Tumor Malignancy

**Figure 1:**
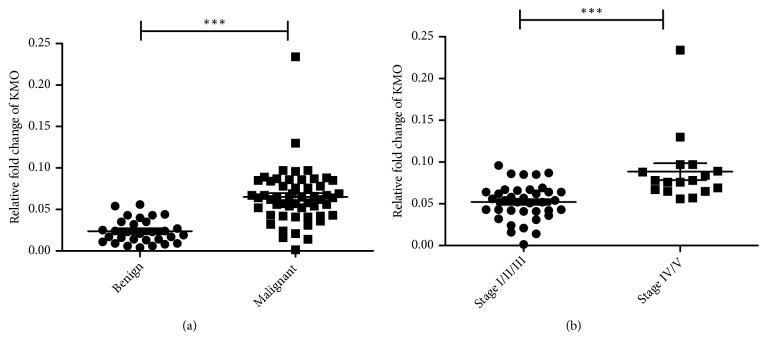
*Comparison of the KMO gene expression in CMTs.* (a)* KMO* gene expression in benign (*n* = 30) and malignant CMT tissues (*n* = 54). (b)* KMO* gene expression in canine malignant CMTs at stages I/II/III (*n* = 37) and stages IV/V (*n* = 17). (*∗∗∗P *< 0.0001).

*KMO* gene expression in clinical CMT specimens was first identified in 84 cases using real-time PCR. Interestingly, significantly higher expressions of* KMO* (*p* < 0.0001) were observed in malignant CMTs than in benign CMTs (Figure 1(a)). In addition, the* KMO* gene (*p *< 0.0001) was overexpressed in stage VI/V CMTs (Figure 1(b)). The data showed that* KMO* gene expression discriminated dogs with malignant CMTs from dogs with benign CMTs and indicated that the expression level of the* KMO* gene may provide valuable information for the diagnosis of malignancy and metastasis in canine CMTs.

### 3.2. The Correlation between the Expressions of KMO and Indoleamine-2,3-Dioxygenase Genes in CMTs

**Figure 2:**
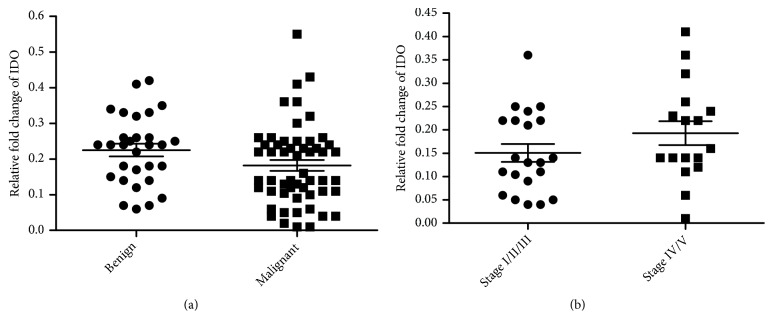
*Expression level of the IDO gene in CMTs.* (a)* IDO* gene expression in benign and malignant CMT tissues. (b)* IDO* gene expression in canine malignant CMTs at stages I/II/III and stages IV/V.

Indoleamine-2,3-dioxygenase (IDO) is located upstream of KMO in the kynurenine pathway [46]. We therefore sought to clarify whether the overexpression of* KMO* was related to the* IDO *expression. The results showed that there was no significant difference in* IDO* expression between malignant and benign CMTs or between CMTs with or without metastasis (Figures 2(a) and 2(b)). These findings indicated that* KMO* overexpression was not* IDO*-dependent in CMTs.

### 3.3. The Correlation between KMO Protein Expression and CMT Malignancy

To further determine the association between KMO and tumor progression, KMO expression in CMTs was analyzed by immunohistochemistry and scored by immunoreactive scoring (IRS) under the conditions listed in Table 1: Primers for canine* IDO*,* KMO*, actin, and* HPRT.*

**Figure 3:**
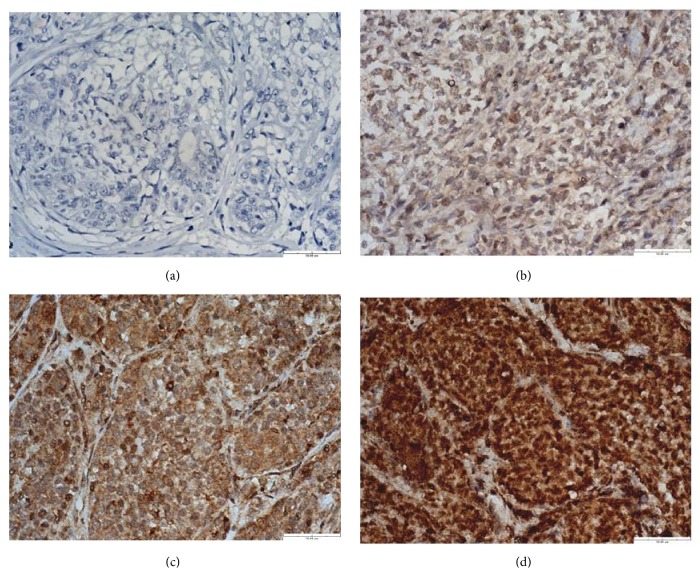
*Immunohistochemical analysis of KMO protein expression in CMTs.* (a) CMT stained without antibody against KMO as a negative control, which did not show immunoreactivity in the cytoplasm. (b) CMT with weak KMO cytoplasmic staining (1+). (c) CMT with moderate KMO cytoplasmic staining (2+). (d) CMT with strong KMO cytoplasmic staining (3+). Scale bar = 50.00 *μ*m.

**Figure 4:**
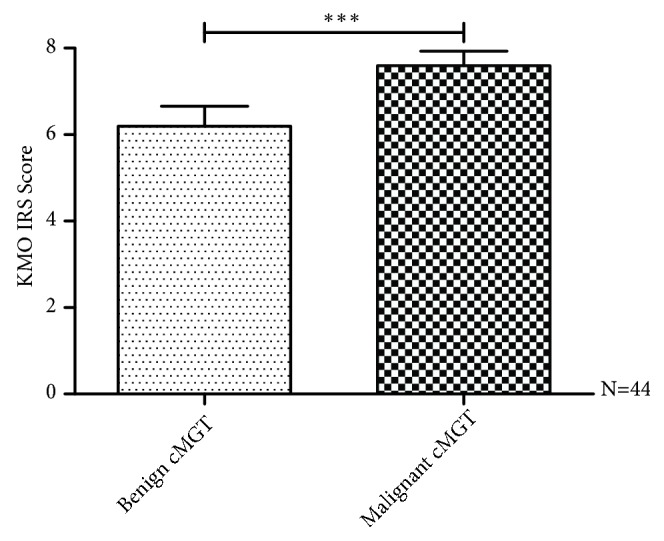
*Correlation between KMO IRS and pathologic malignancy in CMTs.* The expression of the KMO protein was analyzed by immunohistochemistry and scored by immunoreactive score (IRS). KMO IRS showed a statistically significant association with tumor malignancy (*P *< 0.05). KMO IRS in malignant CMTs was significantly higher than that in benign CMTs (*∗∗∗P < *0.001).

Table 2 shows that the standards for scoring KMO protein are shown in Figure 3. According to the IRS, KMO expression could be classified into three groups. Thus, tumors were identified as KMO negative (IRS 0-3), weak (IRS 4-6), and strong (IRS 7-9). Further analysis showed that the level of KMO expression was significantly associated with ovariohysterectomy (OHE) status; 21/39 (53%) patients with a strong KMO expression had OHE prior to the surgery to remove tumors (*p* < 0.05). The level of KMO expression was also significantly associated with tumor malignancy, tumor size, and tumor recurrence. In total, of 39 CMTs with a strong KMO expression, 27/39 (69%) tumors were malignant (*p *< 0.001). The correlations between the level of KMO expression and the characteristics of the patients with CMTs are summarized in Table 3. Moreover, as shown in Figure 4, KMO IRS in malignant CMTs was significantly higher than that in benign CMTs (*p < *0.001). These results suggested that the KMO level can be used to discriminate malignant CMTs from benign tumors.

### 3.4. The Association between KMO Expression and the Survival Time in CMT Patients

**Figure 5:**
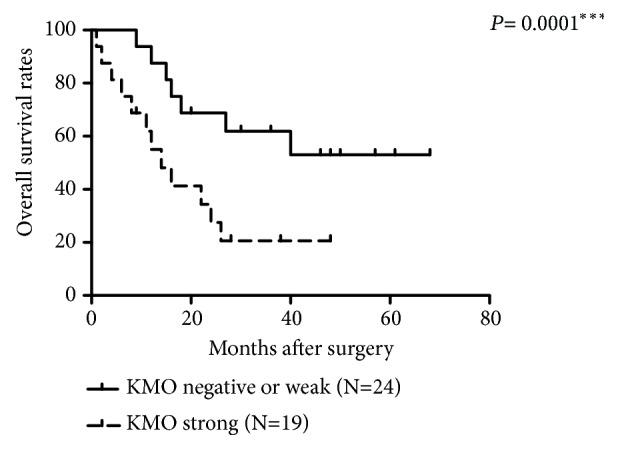
*Association between KMO expression and survival time of CMT patients.* Patients with KMO strong staining tumors had a significantly shorter survival time and a remarkably lower survival rate than those with KMO negative or weak tumors (*∗∗∗P *< 0.001).

Because tumor malignancy determines the survival outcome of cancer patients, we next evaluated the association between KMO expression and the overall survival rate of dogs with CMTs. The KMO expression could be classified into three groups according to the IRS. The Kaplan–Meier survival curves showed that patients with strong KMO-expressing tumors had a significantly shorter survival time and a remarkably lower survival rate than those with negative or weak KMO-expressing tumors (*p* < 0.001) (Figure 5). Taken together, the results shown here were similar to the profile of the* kmo* gene, demonstrating that KMO is a potential biomarker for predicting the prognosis of CMT dogs.

### 3.5. The Role of KMO in the Proliferation of CMT Cells

**Figure 6:**
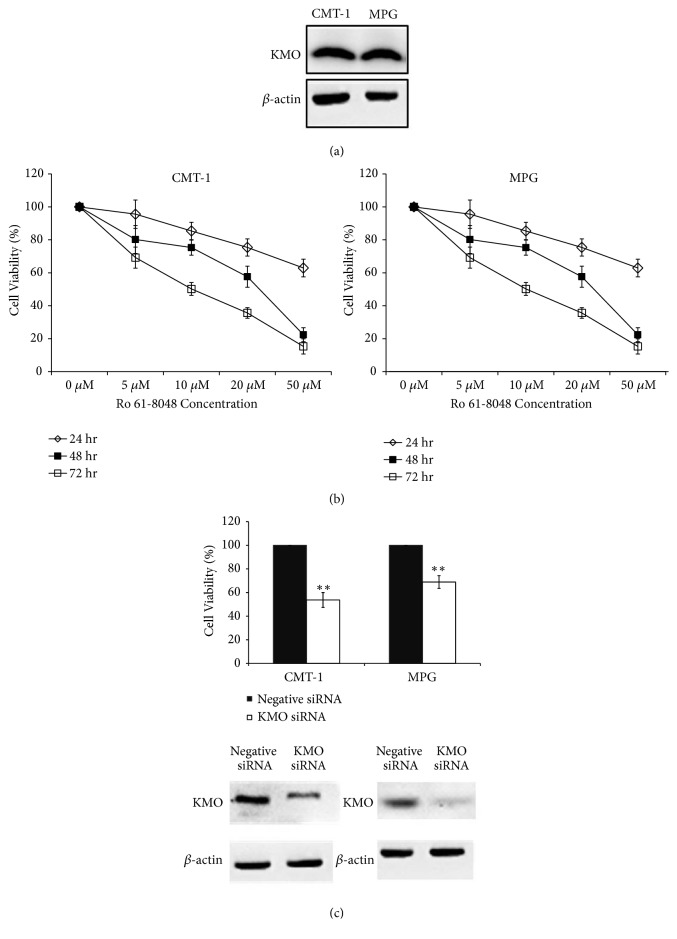
*Downregulated KMO activities with KMO inhibitor or siRNA inhibited cell proliferation in CMT cells.* (a) KMO expression in CMT-1 and MPG cells. (b) Cells treated with Ro 61-8048 for 24, 48, and 72 hrs were found to exhibit significantly suppressed cell proliferation of CMT-1 and MPG cells. (c) Knockdown of* KMO* with siRNA reduced cell proliferation in comparison to cells with control siRNA treatment.* Columns*, mean;* bars*, SD (*n* = 3). *∗∗P *< 0.01.

High KMO expression was proved to be associated with the malignancy of CMT and indicated a poor outcome of the patients. The role of KMO in CMT development was next verified. We first examined the KMO expression in CMT cell lines (CMT-1 and MPG cells) and found that both had identifiable KMO protein amounts (Figure 6(a)). Incubation of CMT-1 and MPG cells with a KMO inhibitor (Ro 61-8048) for 1~3 days significantly inhibited cell proliferation (Figure 6(b)), and similar results were also observed when silencing KMO expression with specific siRNAs against* KMO* (Figure 6(c)). The data suggested that KMO might play an important role in CMT cell growth.

## 4. Discussion

CMT is the most frequently diagnosed type of cancer in female dogs [47, 48], and approximately half of CMTs are malignant [49]. Surgical excision is the most effective treatment for CMT, but dogs with CMT have around a 30-58% recurrence or metastasis rate within 2 years following surgical removal [49, 50], and about 40-60% die from cancer-related diseases within the first 2 years [51]. The low survival rate of patients implies a low rate of specific diagnoses and ineffective therapies in CMT treatment. Challenges of CMT treatment include complex histological classification as well as unpredictable tumor behavior and prognosis [52]. Therefore, it is necessary to improve the accuracy of diagnosis to facilitate the determination of appropriate therapies.

Herein, we identified KMO as a novel and potential biomarker in CMT, which can help to improve diagnosis and predict the prognosis of CMTs. Our results showed that 31.8% of the total CMTs and 73.7% of the malignant CMTs had strong expressions of KMO protein (Table 3). This indicated that the expression of KMO protein issignificantly associated with tumor malignancy and demonstrated the potential of KMO in discriminating malignant tumors from benign ones. Furthermore, the survival rate of patients with a strong KMO protein expression was lower than that of those with weak or negative KMO expression. This result suggested that KMO could be a promising biomarker not only for tumor malignancy but also for predicting the prognosis of CMT patients.

KMO is involved in the metabolism of tryptophan and catalyzes the conversion of kynurenine into 3-HK and 3-hydroxyanthranilic acid, which are further converted into quinolinic acid, generating NAD^+^ for essential cell survival [53]. The kynurenine pathway involves physiological and pathological processes in the nervous and immune systems. KMO is notable because it has been proven to be a potential therapeutic target for stroke, seizures, and Huntington's disease [54]. IDO is located upstream of KMO in the kynurenine pathway [43]. The potential association between IDO expression and cancer has been intensively studied [55]; however, their relationship is still ambiguous and sometimes controversial. Reports have shown that IDO overexpression in human tumors is related to tumor growth, but other reports have suggested that IDO expression in tumor cells and antigen-presenting cells inhibits tumor proliferation [55]. In our results,* KMO *overexpression was independent of* IDO *expression in CMTs, suggesting that KMO might induce tumor malignancy via a novel mechanism not involving IDO.

We also demonstrated that knockdown of KMO expression or blocking of its activity could suppress proliferation of CMT cells. Herein, we found that though CMT-1 and MPG are both cell lines of canine mammary gland tumors, CMT-1 is developed from canine mammary carcinoma (epithelial cell origin) while MPG is derived from the canine mixed mammary gland tumor. The different cell origins of CMT-1 and MPG may have relied on differently growth signal pathways and therefore have different sensitivities to KMO knockdown. KMO has been reported to play a role as an agonist for the N-methyl-D-aspartic acid (NMDA) receptor [56]. NMDA receptors are known to initiate gene activation and cell proliferation and promote cell survival via the extracellular signal-regulated kinase (ERK1/2) pathways [57]. Recently, a report showed that NMDA receptors were overexpressed in human breast cancer cell lines [58]. Another metabolite of kynurenine produced by the action of KMO, 3-hydroxyanthranilic acid, causes apoptosis of Th1 cells by activating caspase-8 [59] and induces apoptosis of T-cells through the inhibition of NF-*κ*B [60]. Taken together, although the detailed mechanisms still need to be fully elucidated, our results have offered significant evidences of involvement of KMO in CMT progression and provided precious advice for further study on human breast cancer therapy.

## 5. Conclusions

A significant parallel increase of KMO mRNA and protein expression in malignant CMT was revealed and correlated with shorter survival time in CMT patients. Our results also showed that KMO plays a role in controlling cell growth and malignancy in canine mammary tumors. These findings indicate the potential applications of KMO in cancer prognosis and therapeutic developments.

## Figures and Tables

**Table 1:** Primers for canine *IDO*, *KMO*, actin, and *HPRT*.

Gene	Forward	Reverse
*IDO*	CAGCTCACCGGGACTTTCTT	TCCATGGCATTAGTGCCTCC
*KMO*	ATGGAGTCATCAGACGTTCA	GTGACCCCATGGAGTTTGCA
Actin	CGACCTGACCGACTACCTCA	TTTGATGTCACGCACGATTT
*HPRT*	TGCTCGAGATGTGATGAAGG	TCCCCTGTTGACTGGTCATT

**Table 2:** Classification of KMO expression as determined by immunoreactive score (IRS).

Intensity of immunoreactivity	Score	Proportion reactive	Score
No staining	0	No staining	0
Weak cytoplasmic staining	1	< 10%	1
Moderate cytoplasmic staining	2	10%-50%	2
Strong cytoplasmic staining	3	> 50%	3

**Table 3:** Characteristics of the patients correlated with expression of KMO protein.

Characteristics		KMO		*P* value
	Negative 0-3	Weak 4-6	Strong 7-9	
*All patients*	6/ 86 (7%)	41/86 (48%)	39/86 (45%)	
*Age*				
<9 years	4/6 (67%)	12/41 (29%)	6/39 (15%)	
≥ 9 years	2/6 (33%)	29/41 (71%)	33/39 (85%)	0.271
*Tumor size*				
≤ 5 cm maximum diameter	4/6 (67%)	31/41 (76%)	25/39 (64%)	
> 5 cm maximum diameter	2/6 (33%)	10/41 (24%)	14/39 (36%)	*0.028∗*
*Ovariohysterectomy status*				
No	5/6 (100%)	37/41 (90%)	18/39 (47%)	*0.031∗*
Yes	1/6 (0%)	4/41 (10%)	21/39 (53%)	
*Malignancy*				
Benign	6/6 (100%)	31/41 (76%)	12/39 (31%)	
Malignant	0/6 (0%)	10/41 (24%)	27/39 (69%)	*0.0004∗∗*
*Tumor stage (N=37)*				
I, II and III	---	3/10 (30%)	11/27 (41%)	
IV and V	---	7/10 (70%)	16/27 (59%)	*0.046∗*
*Lymph node metastasis *				
No	---	7/10 (70%)	16/27 (59%)	0.208
Yes	---	3/10 (30%)	11/27 (41%)	
*Distant metastasis*				
No	---	8/10 (80%)	24/27 (89%)	0.951
Yes	---	2/10 (20%)	3/27 (11%)	
*Recurrence*				
No	6/6 (100%)	19/41 (46%)	9/27 (33%)	
Yes	0/6 (0%)	22/41 (54%)	18/27 (67%)	*0.025 * **∗**

*∗P* < 0.05; *∗∗P* < 0.01.

## Data Availability

The data used to support the findings of this study are available from the corresponding author upon request.
